# Ultrasound-guided sclerotherapy of pingyangmycin for periorbital lymphatic malformations

**DOI:** 10.1186/s12886-025-04154-0

**Published:** 2025-05-30

**Authors:** Yixiao Li, Liang Wang, Dan Song, Bingxuan Jiao, Jing Li, Jie Zhou, Lei Guo

**Affiliations:** 1 Department of Cheeloo College of Medicine, Shandong University, Jinan, 250001 China; 2 Department of Vascular Anomalies and Interventional Radiology, Children’s Hospital Affiliated to Shandong University, Jinan, China; 3 Department of Vascular Anomalies and Interventional Radiology, Jinan Children’s Hospital, Jinan, China

**Keywords:** Lymphatic malformations, Periorbital, Sclerotherapy, Ultrasound-guided, Pingyangmycin

## Abstract

**Background:**

This study investigated the treatment outcomes of ultrasound-guided sclerotherapy with pingyangmycin for periorbital lymphatic malformations (LMs).

**Method:**

A retrospective study of patients with periorbital LMs who underwent ultrasound-guided sclerotherapy in our department between 2017 and 2024 was conducted.

**Results:**

One boy and seven girls with periorbital LMs were analyzed. Four cases had intraorbital lesions, two cases had lesions of the eyelids, and two cases had mixed lesions. Eight children underwent 13 sclerotherapy sessions, fluid was able to be withdrawn with a median amount of aspirate of 2 ml. After treatment, one of the eight children was lesion not seen on imaging, six showed strong improvement, and one showed weak improvement. None of the children experienced procedure-related adverse reactions.

**Conclusions:**

Ultrasound-guided sclerotherapy is a treatment modality for periorbital LMs with good efficacy and few potential risks.

## Synopsis

Ultrasound-guided sclerotherapy with Pingyangmycin is a treatment modality for periorbital LMs with good efficacy and few potential risks.

## Background

The incidence of lymphatic malformations (LMs), a slow-flow vascular malformation, is approximately 1.2–2.8 per 1000 births [1]. Periorbital LMs is a rare lesion that invades the orbit, eyelids, and conjunctiva, which account for 46.5% of periorbital vascular anomalies [2]. In addition, 9% of large cervicofacial LMs extend to the orbital region [3]. Most periorbital LMs have intraorbital and periorbital components, and are usually insinuated between normal orbital structures, causing bone remodeling and anomalies [4]. The typical clinical manifestations of periorbital LMs in children include proptosis, ptosis, extraocular motility restriction, swelling, compression of the optic nerve, and amblyopia [3, 5–7].

Treatments for periorbital LMs include surgical resection, sclerotherapy and sirolimus, which have not yet been standardized because of their low prevalence. In this article, we summarize the clinical data of 8 cases of periorbital LMs treated with ultrasound-guided sclerotherapy, with the aim of providing an evidence-based approach for the treatment of periorbital LMs.

## Methods

We retrospectively reviewed patients with periorbital LMs who underwent ultrasound-guided sclerotherapy in our department between 2017 and 2024. The diagnosis of LMs was established based on clinical symptoms and ultrasound (US), and the condition of the LMs was further visualized using magnetic resonance imaging (MRI). This study was approved by the Ethics Committee of Children’s Hospital Affiliated with Shandong University (SDFE-IRB/T-2024023) and written informed consent to participate was obtained from their parents or legal guardians. The patient records included age, sex, lesion side, symptoms, complications, therapy, prognosis, and serious adverse reactions. Children with poor compliance or incomplete data were excluded.

All patients underwent sclerotherapy under general anesthesia performed by experienced interventional radiologists. All sclerotherapy operations were performed under ultrasound guidance, including puncture of the lesion, aspiration of the cystic fluid, and sclerosant injection. And intraoperative care was taken to avoid injecting sclerosant into the normal periorbital tissue outside the lesion. The sclerosant used in this study was pingyangmycin, which was given injection for cystic lesions mixed with dexamethasone and iodixanol (a contrast agent) at a concentration of 2 mg/ml: pingyangmycin 8 mg + dexamethasone 2 mg/1 ml + iodixanol 3 ml after aspiration of cystic fluid. For microcystic lesions, sclerotherapy was carried out infiltrative injection of a low concentration of pingyangmycin (concentration of 0.5 mg/ml: pingyangmycin 8 mg + dexamethasone 2 mg/1 ml + iodixanol 15 ml). The purpose of mixing pingyangmycin with dexamethasone is to minimize potential allergic and other risks, and dexamethasone is unlikely to affect other mechanisms of action of sclerosant [8]. And the maximum pingyangmycin dose should not exceed 8 mg per sclerotherapy session [9]. After the sclerotherapy operation was completed, single-frame radiography was performed to observe whether the contrast agent morphology was consistent with the MRI morphology (Iodixanol was added when preparing the pingyangmycin solution, making it visible during radiography).

All children were routinely administered prophylactic hemostatic medication (“Hemocoagulase Atrox for Injection” at a dosage of 0.25–0.5 IU) for two days postoperatively, and symptomatic treatments for swelling, dry eye, and eye irritation were administered when necessary. If additional treatment was required, the procedure was repeated in appropriately 4–6 weeks.

The evaluation of treatment response in this study was accomplished primarily through objective imaging evidence, such as US or MRI comparisons before and after treatment. It was conducted by a professional doctor who did not participate in this study. An overall evaluation percentage (0% = no change to 100% = lesion not seen on imaging; improvement in volume was considered weak between 0 and 20%, moderate between 20 and 50%, and strong when ≥ 50% was recorded [10].

## Results

A total of 1140 children with LMs were recorded, including 511 in the head and neck. After careful screening, a total of 9 children with periorbital LMs which received ultrasound-guided sclerotherapy had clinical data. However, one patient had extensive maxillofacial LMs with eyelid involvement and received sclerotherapy for the maxillofacial LMs only, without treatment of the periorbital LMs, and was excluded.

In total, 8 cases (1 boy and 7 girls) with periorbital LMs were analyzed, the median age at presentation was 5.5 years (range: 2 months − 11 years)with a median weight of 21 kg (range: 11.5–43 kg). Six cases involved the right side and two involved the left side. And Four cases were intraorbital lesions, two cases were lesions of the eyelids, and two cases were mixed lesions. Six children presented suddenly and had a short duration of illness, whereas two had a long duration of illness. All children presented with localized swelling, in addition to proptosis in 3 cases, conjunctival congestion/edema in 2 cases, optic nerve compression in 2 cases, ptosis in 1 case, difficulty opening the eyes in 1 case, ocular compression in 1 case, and a combination of decreased visual acuity and blurred vision in 1 case. Table 1 lists the treatment details of the patients.

**Fig. 1:**
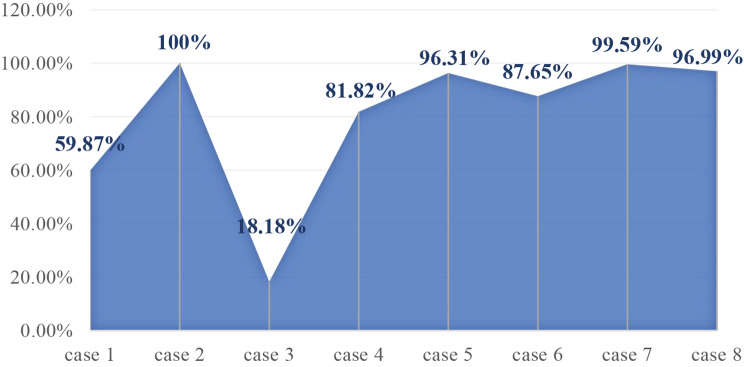
Volumetric response rate with periorbital LMs

Eight children received 13 sclerotherapy sessions (5 cases received 1 session, 1 case received 2 sessions, and 2 cases received 3 sessions). During sclerotherapy, the lymphatic fluid of all patients was aspirated under ultrasound guidance with a median return of 2 ml (range: 0.5–10 ml). Notably, in the evaluation of efficacy with postoperative imaging (Fig. 1), 1 case was lesion not seen on imaging after treatment, 6 cases showed strong improvement, and 1 case showed weak improvement. None of the children experienced procedure-related adverse reactions.

**Table 1:** Demographic characteristics, clinical features, and treatment in children with periorbital LMs

Case	Demographic characteristics			Subtypes	symptoms of presentations		previous treatment	Intraoperative fluid volume(ml)	Efficacy evaluation			
	Sex	Years	Medical history		Reason for referral	Accompanying symptoms			Initial dimensions of lesion (cm)	Final dimensions of lesion(cm)	Follow up	Outcome
1	F	25 M	15 D	intraorbital + eyelid	intracystic hemorrhage	swelling	none	2	1.3 × 2.3	0.6 × 2	6.5 M	strong
2	F	10Y	7 D	intraorbital	intracystic hemorrhage	swelling, conjunctival congestion, ocular compression	none	2	2.9 × 2.5 × 1.9 (Fig. 2A)	0 (Fig. 2B)	2.5 M	lesion not seen on imaging
3	F	3Y	2 M	intraorbital	intracystic hemorrhage	swelling, ptosis, optic nerve compression	none	5	2.8 × 3.5 × 2.3	2.0 × 2.2 × 1.6	1 M	strong
4	F	11Y	11 Y	eyelid	lesions	swelling	none	0.5	1.1 × 0.6 × 0.5	0.9 × 0.6 × 0.5	1 M	weak
5	F	7Y	5 Y	intraorbital + eyelid	intracystic hemorrhage	swelling	none	3(1st ), 1(2nd ), 1(3rd )	3.2 × 2.2 × 2.1	1.3 × 0.7 × 0.6	1.5 M	strong
6	F	14 M	1 M	intraorbital	intracystic hemorrhage	swelling, proptosis, conjunctival edema, difficulty opening eyes	none	10(1st ), 2(2nd )	2.4 × 1.7 × 1.5	1.8 × 0.7 × 0.6	3 W	strong
7	M	4Y	7 D	intraorbital	intracystic hemorrhage	swelling, proptosis, optic nerve compression, decreased visual acuity, blurred vision	none	0.5(1st ), 0.5(2nd ), 0.5(3rd )	2.5 × 1.8 × 1.3	0.4 × 0.3 × 0.2	4.5 M	strong
8	F	11Y	20 D	eyelid	intracystic hemorrhage	swelling	none	2	3.1 × 2.0 × 1.8	1.4 × 0.6 × 0.4	2.5 M	strong

*Note* M: male, F: female, Y: year, M: month, W: week, intraorbital: lesions within the orbit (intra- and extra-conal), Follow up: the time interval between last procedure and the follow up imaging

**Fig. 2A:**
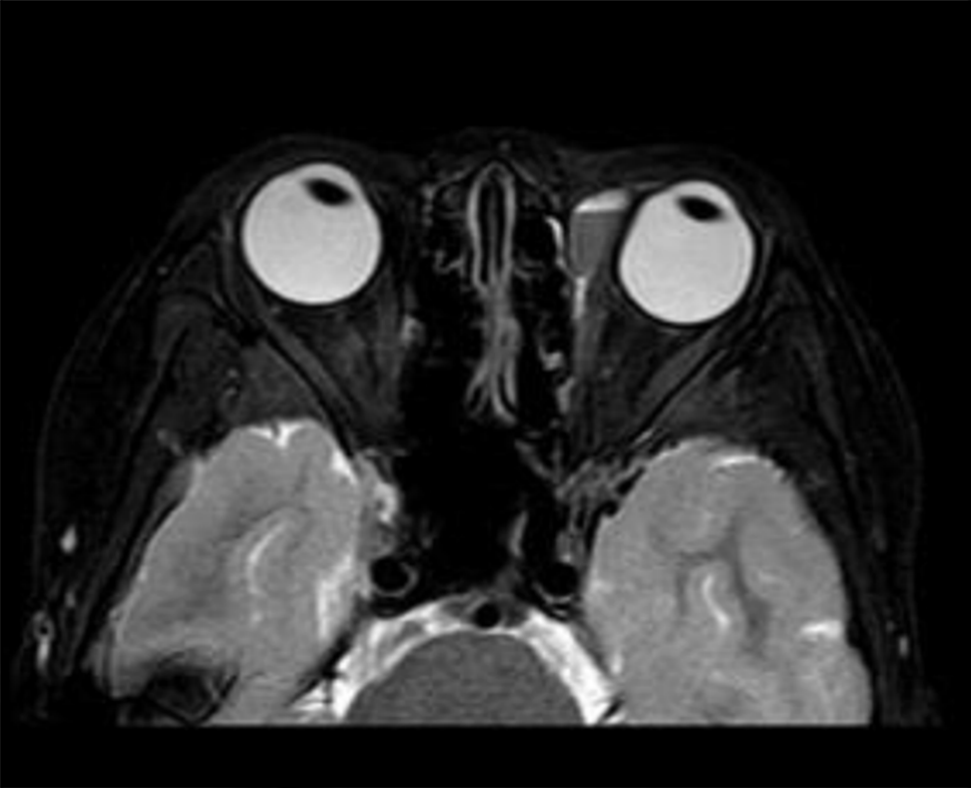
A 10-year-old girl presented with a left periorbital LMs with intracystic hemorrhage on MRI, accompanied by mild compression of the globe

**Fig. 2B:**
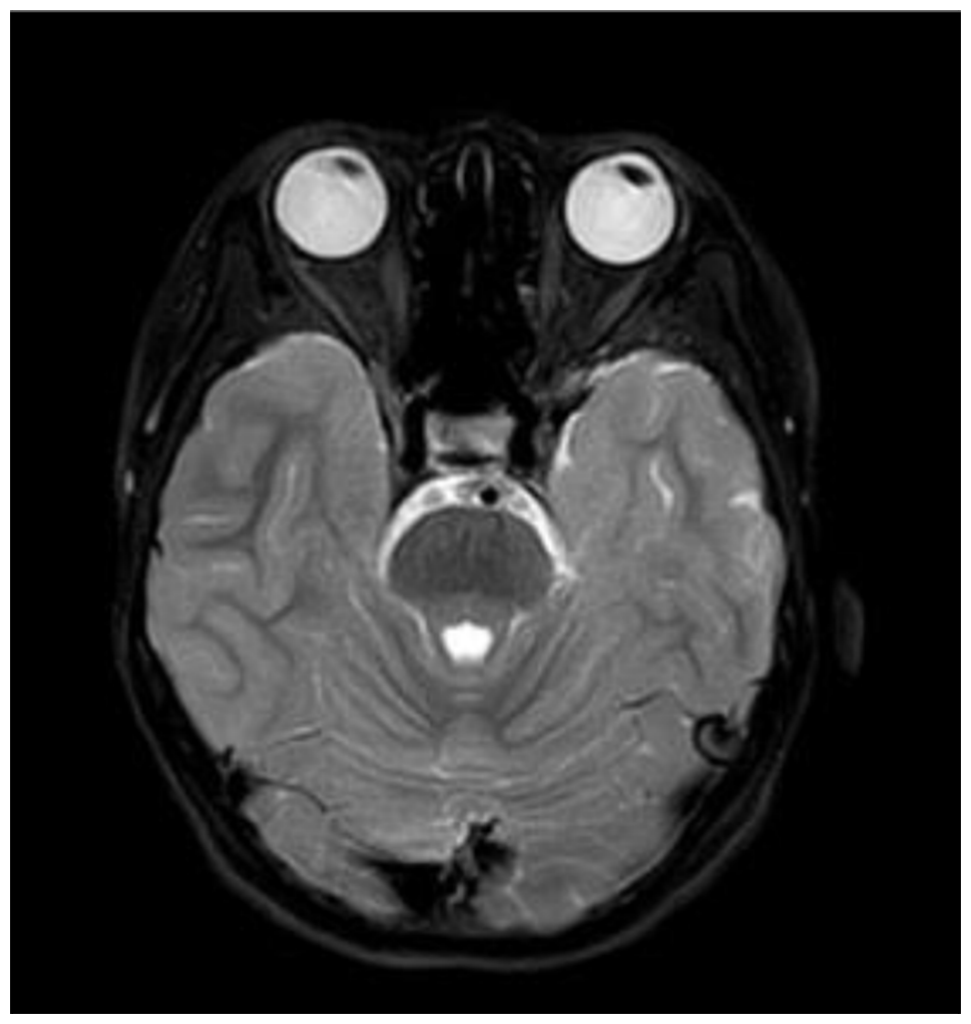
The lesions disappeared after one course of sclerotherapy

## Discussion

Observation is acceptable for periorbital LMs with mild, nonprogressive symptoms without any functional impairment [5, 11]. However, owing to intracystic hemorrhage, venous thrombosis, inflammation, or proliferation of the internal lymphoid tissue, LMs may abruptly enlarge and cause pain, swelling, proptosis, ocular dysmotility, acute visual loss (due to hemorrhage or thrombosis and optic nerve compression), or even keratopathy [7, 12]. In our study, all children presented with periorbital swelling and 7 cases had LMs combined with intracystic hemorrhage. Other manifestations included ptosis, proptosis, ocular compression, difficulty in opening the eyes, optic nerve compression, and loss of visual acuity, which are generally consistent with previous reports.

Surgical excision of LMs remains an option and should be tailored according to the location, depth, and relationship between the periorbital structures and vascular structures [13–15]. However, it is often not recommended because of its irregular, non-encapsulated, infiltrative nature and proximity to surrounding structures [16]. Compared to surgical resection, sclerotherapy is not only a less invasive therapeutic option, but also a faster recovery [5, 17]. A meta-analysis of 13 studies and a total of 154 patients showed that after sclerotherapy, the rate of complete cure was 54.9%, emergency decompressive surgery was 3.4%, and vision loss was 2.7% [18]. Although the sample size was relatively small in our study, the eight children with LMs in this study underwent a total of 13 sclerotherapy sessions, which showed a significant improvement rate of 87.5% (7/8).

Bleomycin is one of the most widely used in the sclerotherapy of LMs [19], and it was first used as a sclerosant for periorbital LMs in 2012 [20]. Intralesional bleomycin injection is an effective treatment for inducing fibrosis and facilitating the surgical debulking of periorbital LMs [21]. Among patients with periorbital LMs who received bleomycin sclerotherapy, 93% achieved excellent or good results [22]. Pingyangmycin, was also known as bleomycin A5, was first reported for the clinical treatment of vascular malformations in 1991 in China [9]. It has been reported to be as effective as bleomycin with the advantage of fewer complications [11]. In this study, pingyangmycin was used in all cases. In addition to achieving a relatively favorable prognosis, no significant drug-related adverse effects were observed in any child.

The main challenge in sclerotherapy for periorbital LMs is to ensure that it safely accesses the retroorbital space [23]. US has emerged as an invaluable tool during sclerotherapy [24], and using ultrasound to precisely guide the injection should also be considered main factor for the encouraging outcome in this study. LM presents on ultrasound as ill-defined and mostly anechoic lesions. In cases of recent hemorrhage or infection, US imaging may be more heterogeneous, and fluid-to-fluid levels may have developed [12]. Notably, excessive probing and manipulation should be avoided during sclerotherapy [23].

Ipsilateral intracranial developmental venous anomalies have been reported to be associated with periorbital LMs [3, 25]. In an imaging study involving 33 patients with periorbital LMs, 70% of the patients had different types of intracranial vascular malformations [26]. However, in this retrospective study, no information was queried regarding documentation.

## Conclusion

Periorbital LMs seriously affect the ocular health of children. Ultrasound-guided sclerotherapy is feasible for the management of periorbital LMs with good efficacy and few potential risks.

## Data Availability

All data generated or analysed during this study are included in this published article.

## Abbreviations

LMs: Lymphatic Malformations
US: Ultrasound
MRI: Magnetic Resonance Imaging
